# Optimized Tribological Performance of Nitrogen-Doped Diamond-like Carbon Films on NBR: Influence of Bias Voltage of DC Magnetron Sputtering

**DOI:** 10.3390/nano14070564

**Published:** 2024-03-24

**Authors:** Tao Yang, Changxin Han, Qiaoyuan Deng, Feng Wen

**Affiliations:** Special Glass Key Laboratory of Hainan Province, School of Materials Science and Engineering, Hainan University, Haikou 570228, China; 19080500110014@hainanu.edu.cn (T.Y.); hcx0919@hainanu.edu.cn (C.H.); qydeng@hainanu.edu.cn (Q.D.)

**Keywords:** NBR, DLC films, nitrogen-doped, bias voltage, tribological properties

## Abstract

In this research, nitrogen-doped diamond-like carbon (N-DLC) coatings were deposited on Nitrile Butadiene Rubber (NBR) substrates using direct current magnetron sputtering (DC-MS) under varying bias voltages. This study aimed to explore environmentally friendly, low-wear, and non-lubricating seal coatings to enhance the durability of rubber sealing products, which predominantly operate under dynamic sliding conditions. By reducing the coefficient of friction (CoF), the friction and wear on rubber products can be significantly minimized, extending their lifespan. This investigation thoroughly examined the microstructure, mechanical properties, and tribological behavior of the N-DLC films. Among the coatings, the one produced at a bias voltage of −50 V demonstrated superior hardness, elastic modulus, and the lowest CoF in comparison to those prepared with 0, −100, and −200 bias voltages. This optimal combination of properties resulted in an exceptionally low wear rate of 10^−9^ for the film deposited at −50 V, indicating its outstanding wear resistance.

## 1. Introduction

NBR, a synthetic rubber copolymer derived from butadiene and acrylonitrile, boasts high resistance to oils, fuels, and other petroleum-based substances, rendering it ideal for seals, gaskets, O-rings, and hoses in automotive and machinery applications [1]. Its exceptional sealing properties, coupled with its resistance to oils and fuels, have cemented its popularity in engines, hydraulic systems, and various machinery components [2]. However, despite its numerous merits, NBR is frequently utilized in seals and gaskets where wear resistance is paramount. Given that these components endure repeated compression and movement and are challenging to replace frequently, enhancing the service life of NBR is imperative [3,4]. Consequently, the wear resistance of NBR plays a pivotal role in ensuring an extended service life.

DLC (diamond-like carbon) films, consisting of materials exhibiting properties akin to diamond, are thin coatings valued for their hardness, wear resistance, and low friction across diverse industrial and technological realms [5,6,7,8,9,10]. Typically amorphous, DLC films comprise a blend of sp^2^ (graphite-like) and sp^3^ (diamond-like) carbon bonds, endowing them with diamond-like characteristics [11]. DLC films, known for their diamond-like hardness, have excellent wear and abrasion resistance, making them ideal for durable applications. Their hardness and low CoF often lead to their use in enhancing surface wear resistance, thereby prolonging component lifespan and minimizing maintenance or replacement needs. Moreover, their chemical compatibility with rubber, owing to their shared carbon composition, coupled with their adjustable properties and diverse structures, positions them as optimal materials for fortifying the wear resistance of rubber surfaces [12].

While the surface modification of rubber with DLC films offers numerous advantages, there are also some potential drawbacks and challenges associated with this process. DLC coatings present a few limitations: (1) high residual stress, (2) poor thermal stability, and (3) inevitable pinhole-like defects [13]. Elemental doping has become a notable method for addressing these issues. Nitrogen doping, in particular, has shown promise in enhancing the hardness and wear resistance of the coating. Nitrogen incorporation can alter the microstructure of the DLC film, resulting in improved mechanical properties and heightened resistance to wear and abrasion [14,15,16]. Additionally, as per findings from our previous study [17], nitrogen doping can augment the adhesion of the DLC coating to the NBR substrate. Enhanced adhesion is crucial for ensuring the durability and long-term performance of the coating. N-DLC coatings demonstrate potential in various practical applications owing to their robust mechanical performance and favorable tunability in terms of structure and morphological features.

Magnetron sputtering stands out as a favored technique for depositing carbon-based films owing to its versatility, precise control over film properties, and scalability [18]. Key processing parameters encompass bias voltage, temperature, working gas species, and pressure [19]. Among these factors, bias voltage holds significant sway over the energy of ions bombarding the substrate surface, thereby exerting a pivotal influence on the properties of the deposited film. This influence can be effectively harnessed to adjust adhesion and overall performance by modulating the sp^2^/sp^3^ ratio and controlling the microstructure. An elevated bias voltage typically corresponds to heightened ion energy, facilitating the nucleation and growth of crystalline phases albeit potentially introducing more defects and stress into the film [20]. An optimal sp^3^C fraction, particularly for hydrogen-free DLC coatings, is attainable within a bias voltage range of 100–200 V [21]. Similarly, a-C:H coatings characterized by high hardness and adhesion can be achieved through a meticulous control of bias voltage in radio frequency-based plasma-enhanced chemical vapor deposition (PECVD) [22]. Notably, bias voltage does not exert a distinct influence on the nitrogen content of films [23]. However, nitrogen doping prompts alterations in the sp^2^C/sp^3^C ratio and surface C/O ratios [24]. These variations in bond fractions significantly impact the hardness, CoF, and wear resistance of DLC coatings.

In the present investigation, N-DLC films were deposited directly onto NBR substrates using DC-MS. This study comprehensively examined the impact of varying bias voltages during the film deposition process on the surface morphology, structural characteristics, wettability, mechanical attributes, and tribological performance of N-DLC films. Additionally, the investigation succinctly elucidated the frictional interaction mechanisms of N-DLC films against rubber materials. The findings provide novel insights into the enhancement of rubber seal surfaces through modification techniques.

## 2. Materials and Methods

### 2.1. Deposition of N-DLC Films

The N-DLC coatings were applied to NBR substrates via reactive magnetron sputtering within an atmosphere composed of argon and nitrogen gases. Prior to the coating process, the rubber substrates were subjected to a meticulous cleaning regimen. This commenced with the application of a detergent solution, followed by submersion in boiling water for a duration of three minutes. The subsequent phase involved ultrasonic cleansing in anhydrous ethanol for ten minutes, this process was repeated in cycles until the ethanol solvent remained colorless post cleansing, indicating the removal of impurities such as dirt and wax. These preparatory steps were crucial for enhancing the adhesion between the deposited film and the rubber substrate [25]. To conclude the cleaning process, the rubber samples were dried using a hairdryer set to blow cold air, preparing them for the deposition of the N-DLC films.

**Figure 1:**
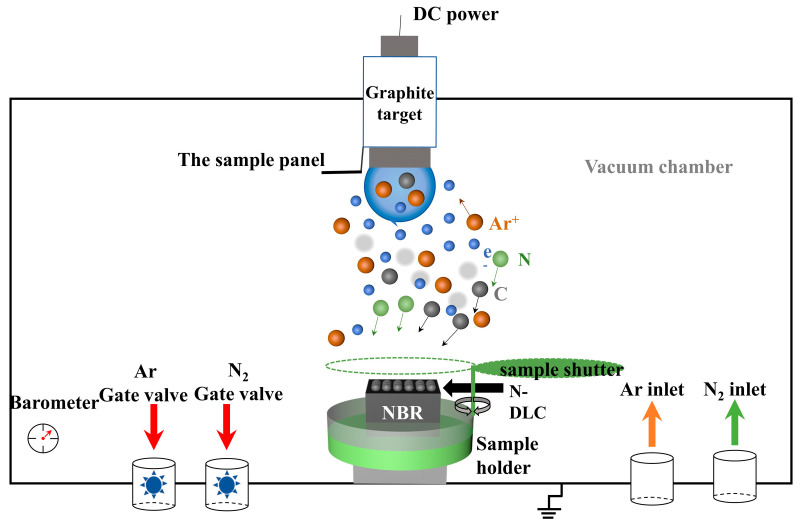
Schematic of the equipment for the preparation of N-DLC films via magnetron sputtering.

The schematic representation of the apparatus utilized for the synthesis of N-DLC films via magnetron sputtering is depicted in Figure 1. This setup comprises a high-purity graphite target (φ76.2 mm × 4 mm, 99.99% purity) positioned within a sputtering chamber that achieves a base vacuum of 3.0 × 10^−3^ Pa. The sputtering process was powered at 100 W, employing a frequency of 40 kHz with a 75% duty cycle. Argon gas was introduced at a flow rate of 30 sccm to serve as the sputtering medium, while nitrogen gas was fed at a rate of 3 sccm to incorporate nitrogen into the DLC films. The substrate bias voltage was varied across four levels: 0 V, −50 V, −100 V, and −200 V, facilitating the deposition of a series of N-DLC films, designated as V0, V50, V100, and V200, onto NBR surfaces and silicon wafers over a 60 min duration. Prior to film deposition, a two-step pre-treatment involving argon plasma was conducted to ensure optimal film adhesion by removing oxides and impurities from the rubber and target surfaces, respectively. The first step was to clean the rubber surface with 30 sccm argon gas at 3.0 Pa, −600 V bias voltage, 75% duty cycle, and 40 kHz. This process lasted for 15 min. The second step was to clean the carbon target at 1.4 Pa, argon flow rate of 30 sccm, 75% duty cycle, 40 kHz, and 100 W DC power. This process lasted for 10 min. This comprehensive setup and procedural detail are summarized in Table 1, illustrating the meticulous approach to N-DLC film preparation via magnetron sputtering.

### 2.2. Material Characterization

The surface morphologies of the samples were examined utilizing an atomic force microscope (AFM, Nanosurf AG Co., Liestal, Switzerland) in tap mode with a silicon probe (Tap 190Al-G, Nasser (Shanghai) Nanotechnology Co., Ltd., Shanghai, China) across a scan area of 10 μm × 10 μm. For visualizing both the surface and cross-sectional morphologies of the films, as well as the surface morphology of the samples’ test area, post-tribological and bonding assessments and a scanning electron microscope (SEM, Quanta 250, Thermo Fisher Scientific Co., Waltham, MA, USA) were employed. The chemical bonding structure and composition of the DLC films were analyzed through Raman spectroscopy (inVia Reflex, Renishaw Co., New Mills, UK, with a laser wavelength of 514.5 nm) and X-ray photoelectron spectroscopy (XPS, Escalab Xi+, Thermo Fisher Scientific, Waltham Co., MA, USA). Notably, the analysis chamber for the XPS was maintained at a vacuum level of 8 × 10^−10^ Pa, utilizing Al kα-rays (hv = 1486.6 eV) as the excitation source.

The contact angle (CA) measurements were conducted at ambient temperature using a JC2000 contact angle tester, employing the sessile drop method. To minimize the influence of the droplet’s own weight, it was crucial to maintain a small droplet volume, which was controlled at 1 μL. For the assessment of all materials, two liquids were chosen: the non-polar CH_2_I_2_ and polar H_2_O. The CA values were recorded 15 s subsequent to the droplet deposition. The solid surface energy (γs), along with its polar ($\gamma_{S}^{p}$) and dispersion ($\gamma_{S}^{d}$) components of surface energy, were calculated utilizing the Owens–Wendt–Rabel–Kaelble (OWRK) method [26]. For each combination of liquid and surface, measurements were repeated three times to reduce uncertainty.

A Berkovich diamond indenter was utilized for conducting nanoindentation experiments (CETR-UMT, Bruker Co., Karlsruhe, Germany). The applied load was incrementally increased from 0 to a peak load of 1 mN over a duration of 15 s, followed by a creep phase lasting 10 s, and a subsequent unloading phase of 15 s. To account for thermal drift, the procedure included a 45 s stabilization period when the load was reduced to 0.1 mN. To mitigate the influence of residual stress from adjacent indentations, five indentations were executed on each sample across five distinct positional zones, maintaining a separation of 20 μm between successive indentation points.

A diamond-tipped scratch tester with a stylus cone tip curvature radius of 12.5 μm was utilized to assess the adhesion strength of the N-DLC coatings quantitatively. The stylus traversed the coatings with a load incrementally increasing from 10 mN to 600 mN over 100 s, at a constant velocity of 20 μm/s. The critical load, marked by an abrupt frictional change, was recorded to gauge the coatings’ adhesive force. For tribological analysis, a CETR-UMT ball-on-disk apparatus from Bruker was employed at room temperature. Zirconia balls with a 3 mm radius were used against the samples, with a corresponding track radius of 6 mm, at 100 rpm and a 0.3 N load for a 60 min test duration. The above test procedure was repeated at least three times to ensure the reliability of the experiment.

(1) $$K=V/\left(F\times L\right)$$

In the context of material wear, the rate of wear, denoted as *K*, is determined by the ratio of the volume of material worn away, represented by *V*, to the product of the applied load, *F*, and the total distance traversed by the moving contact, symbolized by *L*. The calculation of the wear volume *V* is as follows:

(2) V=A×d

The wear track’s cross-sectional area, denoted as *A*, situated beneath the specimen surface’s horizontal line, was quantified using a 3D optical profiler (UP-Lambda, Rtec Co., San Jose, CA, USA). The variable *d* represents the wear track’s length.

## 3. Results and Discussion

### 3.1. Morphology

**Figure 2:**
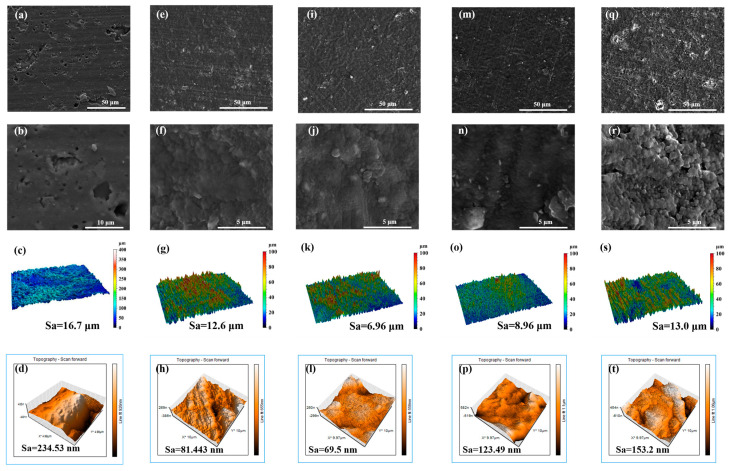
Surface morphologies of NBR and N-DLC films prepared on NBR using different substrate bias voltages. NBR: (**a**–**d**); V00: (**e**–**h**); V50: (**i**–**l**); V100: (**m**–**p**); V200: (**q**–**t**).

To investigate the surface morphology of the film, the authors employed SEM to characterize N-DLC films prepared under varying bias voltages, ranging from low to high, as depicted in Figure 2. In observing the surface morphology of the rubber, it can be found that the rubber injection molding process leaves some perforations and protrusions on the surface of uncoated NBR, which are clearly visible in Figure 2b. The findings indicate that the surface of V50 is notably uniform, exhibiting minimal undulations and stratification, with only a sparse presence of particles. However, with increasing bias voltage, particle agglomeration occurs on the surfaces of films V100 and V200. This phenomenon is likely attributed to the heightened injection energy resulting from the increased bias voltage, leading to the agglomeration of atoms and molecules in the vacuum chamber. Ionized ions, with a smaller mean free path, facilitate sediment agglomeration. Conversely, the film prepared at 0 V displayed a rough morphology due to the absence of negative bias attraction from the substrate. Consequently, it becomes challenging for C and N elements within the cavity to accumulate on the surface of a non-conductive matrix, such as NBR, preventing them from filling the rubber and resulting in defects and voids on the rough surfaces. In contrast, the N-DLC film prepared at −50 V exhibited a relatively smoother surface morphology, attributed to the beneficial effects of an appropriate negative bias. Under the regulation of −50 V, ionized plasma could refine the rough surface of the NBR matrix derived from rolling and stamping [27]. The thicknesses of these sedimentary thin films ranged from about 150 to 180 nm.

In addition, the authors performed macroscopic measurements of the morphology density and roughness of each N-DLC film on NBR as well as on the rubber surface (2 mm × 1.5 mm), as shown in Figure 2c,g,k,o,s. It is worth noting that there is a significant difference between the initial NBR surface and the coated NBR surface. The roughness properties of the surfaces of the film-coated samples were all reduced to varying degrees, with the V50 film having the smallest roughness of 6.96 μm. In contrast, the roughness of V0 was relatively large at 12.6 μm, which is almost comparable to the roughness observed in the V200 samples, which was about 13.0 μm. However, at the macroscopic level, the surface roughness was more affected by the characteristics of the substrate. Given the inherent high roughness of NBR, the roughness of the films may be affected by the substrate and exhibit a corresponding roughness.

In order to investigate the roughness properties of the films in depth, we used atomic force microscopy (AFM) to characterize the micromorphology of each sample, as shown in Figure 2d,h,l,p,t. The surface roughness of V50 remains the lowest at 69.51 nm, while V100 and V200 continue to show higher roughness values and agglomeration. The films prefabricated at 0 V show an inhomogeneous shape and their three-dimensional morphology retains the rift valley configuration, which indicates that the plasma dispersion failed to fill the debris defects in the NBR.

### 3.2. Structure and Composition

**Figure 3:**
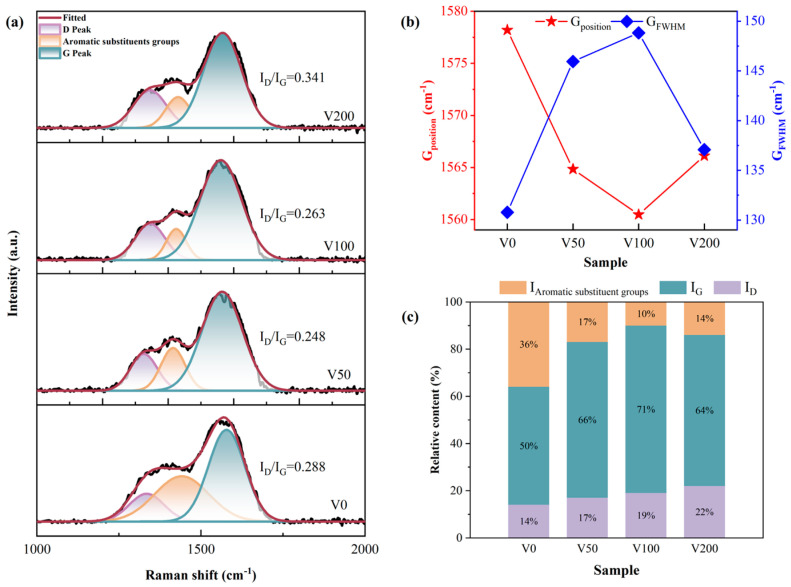
(**a**) Peak fitting of the Raman curves of each sample under different bias voltages to obtain the Raman curves of each sample and their respective I_D_/I_G_ values; (**b**) G peak position and FWHM of each sample; (**c**) peak area fraction of each sub-peak obtained through Gaussian peak fitting.

For single-crystal graphite, a singular Raman peak emerged near 1580 cm^−1^, attributed to the central vibration mode within the optically allowed E_2g_ region of crystallized graphite. This peak, denoted as the G peak [28,29], signifies the presence of sp^2^ C-C hybrid bonds. Conversely, diamond is characterized by a distinct Raman peak at 1322 cm^−1^, which originates from the T_2g_ symmetric vibration mode, signifying the presence of sp^3^ C-C hybrid bonds. The disruption of long-range order leads to the emergence of a peak around 1360 cm^−1^, known as the D peak, which is attributed to phonon dissipation at the boundary of the Brillouin zone [30,31,32]. The emergence of the D peak is indicative of disorder within the sp^2^ hybridized bond angles, suggesting the presence of sp^2^ hybridized carbon structures composed of graphite rings. In all samples depicted in Figure 3, distinct peaks are observable, alongside an additional peak near 1415 cm^−1^, which can be attributed to aromatic substituents in the NBR [4]. During the sputtering process, the temperature within the vacuum chamber was initially at 25 °C ambient temperature and did not exceed 120 °C at the end of deposition process. This temperature is substantially below the degradation temperature of NBR, which is 295 °C [33]. Carbon plasma bombardment on the surface of NBR can induce the random scission of polymer chains, leading to the formation of reactive groups. These groups may undergo internal reactions, migrate to the surface, or engage in reactions with carbon atoms, resulting in the formation of complex cross-linked structures. Additionally, carbon atoms may interact with unpaired sp^2^ bonds present in NBR, facilitating the formation of six-membered rings, thereby reducing the internal energy of the system [34,35]. Typically, the Raman spectrum of a DLC film is characterized by an asymmetric broad peak located near 1500 cm^−1^ [10].

In order to obtain more precise information about the carbon bonding structure in the N-DLC film on NBR, a Gaussian fit was applied to the Raman spectra with baseline subtraction followed by back-convolution, and a sub-peak fit with three peaks is shown in Figure 3a. The qualitative estimation of the sp^3^ hybridization ratio was achieved by calculating the I_D_/I_G_ value from the areas under the D peak and G peak [36]. Generally, an increase in sp^3^ hybrid bonds correlates with a decrease in the I_D_/I_G_ ratio. As illustrated in Figure 3a, with the bias voltage rising from 0 V to −200 V, the I_D_/I_G_ initially decreases and then gradually increases. Alongside the subsurface injection model, low C^+^ incident energy leads to energy consumption through collective atom collisions, hindering the penetration of atoms onto the coating surface. Within a certain range of energy increase, C^+^ penetrates the surface, becoming interstitial atoms in the subsurface layer, thereby increasing local density and compressive stress. The application of compressive stress encourages the atomic groups within diamond-like carbon (DLC) to transition into a stable phase characterized by sp^2^ hybrid bonds. As illustrated in Figure 3b, there is a depiction of the variations in the G peak’s position and its full width at half maximum (FWHM) as a function of the applied bias voltage. An initial increase in bias voltage leads to a broadening followed by a narrowing of the G peak’s half-peak width. Conversely, the peak’s position undergoes the opposite trend. This observation underscores the advantageous impact of elevated bias voltage on the formation of sp^2^ bonds within the nitrogen-doped DLC (N-DLC) film. Such an effect is likely attributable to the high bias voltage facilitating the substitution of carbon atoms with nitrogen within the sp^3^ bond structure, which in turn diminishes the average coordination number from four to three. Furthermore, the higher electronegativity of nitrogen atoms (3.0) in comparison to carbon atoms (2.5) results in an electron cloud distribution that is skewed toward nitrogen atoms. This skewing weakens the carbon–carbon bond and modifies the coordination number from four. This alteration has implications for the stability of the sp^3^ structure and, by extension, influences the hardness of the film [37].

A Gaussian peak fitting analysis revealed the area proportion of each subpeak in Figure 3c. In particular, sample V0 shows a higher proportion of aromatic substituents, potentially due to the rough surface and uneven coverage of the DLC film, making it susceptible to the Raman excitation wavelength of 514.5 nm [38].

**Figure 4:**
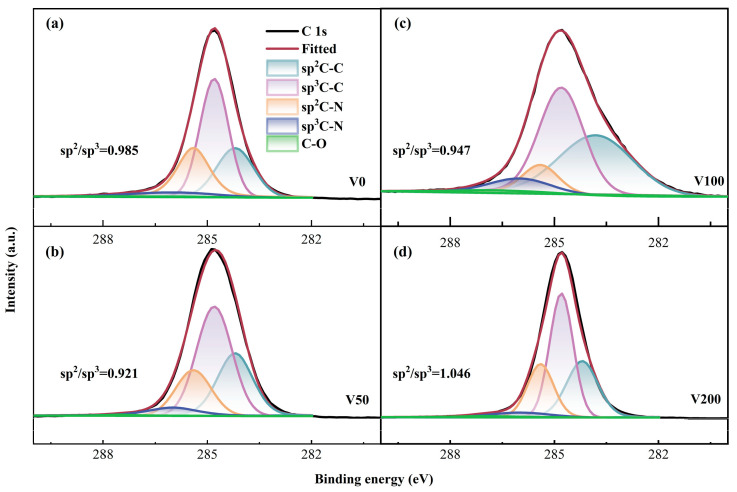
(**a**–**d**) C 1s XPS fine spectrum of as-prepared N-DLC coating.

Moreover, the proportions of sp^2^ and sp^3^ configurations were determined through X-ray photoelectron spectroscopy (XPS) analysis. Figure 4 distinctly displays the significant asymmetry observed in the C 1s spectral line. The integration of nitrogen atoms into the carbon matrix, resulting in the formation of C-N bonds, leads to a shift of the peak position toward higher binding energies. The XPS analysis indicates that nitrogen doping leads to an upshift in the C 1s peak binding energies, attributed to nitrogen’s higher electronegativity compared to carbon. This results in an electron density shift toward nitrogen in the C-N covalent bond. Moreover, the binding energy of the C-N bond, encompassing both sp^2^C-N and sp^3^C-N configurations and is found to be higher than that of the C-C bond, including sp^2^C-C and sp^3^C-C structures. In drawing from the literature [37], the binding energies for the five distinct peak positions are identified as follows: 284.6 eV for the sp^2^C-C bond, 285.2 ± 0.1 eV for the sp^3^C-C bond, 286.1 ± 0.1 eV for the sp^2^C-N bond, 287.4 ± 0.2 eV for the sp^3^C-N bond, and 291.5 ± 0.1 eV for the C-O bond. Additionally, the sp^3^ bond concentration initially increases and then decreases with rising substrate bias, aligning with the I_D_/I_G_ ratio trend observed in the Raman spectroscopy.

**Figure 5:**
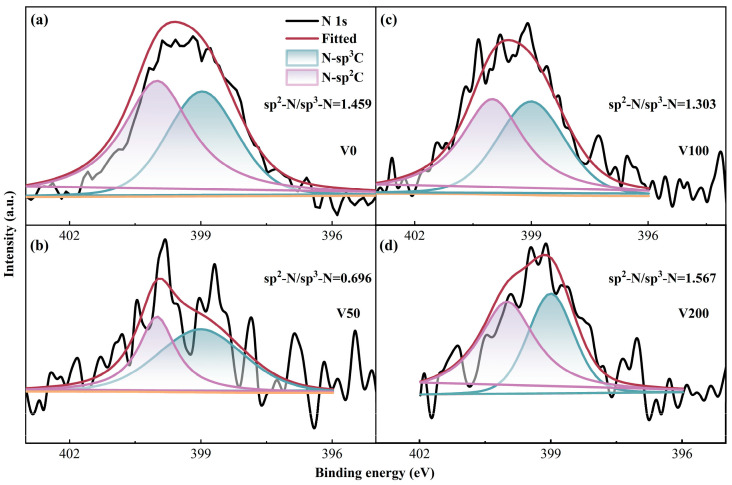
(**a**–**d**) N 1s XPS fine spectrum of as-prepared N-DLC coating.

The initial N 1s spectrum underwent deconvolution, revealing two prominent peaks with binding energies of 398.6 ± 0.1 eV and 399.8 ± 0.2 eV, respectively [39]. The peak at the lower binding energy is attributed to the sp^3^ hybridized C-N bond, while the peak at the higher binding energy corresponds to the sp^2^ hybridized C-N bond. As depicted in Figure 5, the ratio of sp^2^ C-N to sp^3^ C-N initially decreases and then increases with the escalation of substrate bias. This trend indicates that the formation of sp^2^ C-N structures becomes more favorable as the bias voltage increases. This phenomenon can be explained by the differing chemical stabilities between the sp^2^ C-N and sp^3^ C-N bonds.

### 3.3. Contact Angle and Surface Energy

**Figure 6:**
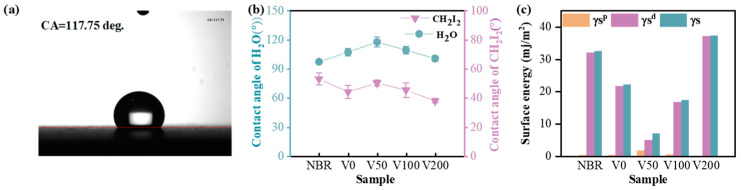
(**a**) Contact angle of V50-H_2_O; (**b**) water contact angle and diiodomethane contact angle of each sample; (**c**) surface energy of each sample.

Figure 6a displays a photograph of the water contact angle (CA) of the V50 sample, indicating a measurement of 117.75 degrees, which is indicative of significant hydrophobicity. Figure 6b depicts the CA change curve in the presence of water and diiodomethane, demonstrating distinct surface wettability characteristics of N-DLC films fabricated under various substrate biases. Notably, both the NBR matrix and the N-DLC film coated on NBR exhibit CA values exceeding 90° when in contact with water, suggesting hydrophobic properties. The curve shows a slight increase in CA with increasing negative substrate bias, peaking at 117 degrees at −50 V. Subsequently, with further negative substrate bias intensification, the water CA begins to decrease but consistently remains higher than that of NBR. Surface roughness generally correlates negatively with CA values, which is consistent with prior roughness observations.

In Figure 6c, the OWRK method was employed to calculate the total surface energy and its polar and dispersion components for both the NBR substrate and the N-DLC film. The surface energy of NBR primarily consists of a single dispersed component. A clear influence of substrate bias on surface energy is evident across all samples. Upon N-DLC film deposition, the total surface energy initially decreases and then increases with rising bias voltage, reaching a minimum of about 7.18 mJ/m^2^ at −50 V. Furthermore, as the bias voltage continues to increase, the surface energy notably rises, which is consistent with previous findings on surface roughness. In all examined samples, including the NBR substrate, the dispersion component ($\gamma_{S}^{d}$) of the surface energy was notably high, often matching or surpassing the total surface energy (γ_S_), with the exception of sample V50, which displayed the lowest surface energy. Notably, the polar component ($\gamma_{S}^{p}$) of surface energy remained minimal across all samples, highlighting that variations in the surface energy of DLC films deposited on NBR are predominantly attributed to the dispersive components rather than the polar components or the total surface energy. This indicates that the dispersion component plays a pivotal role in determining the interfacial wetting behavior of the N-DLC film, particularly in terms of wettability and solid–liquid interactions.

### 3.4. Mechanical Properties

**Figure 7:**
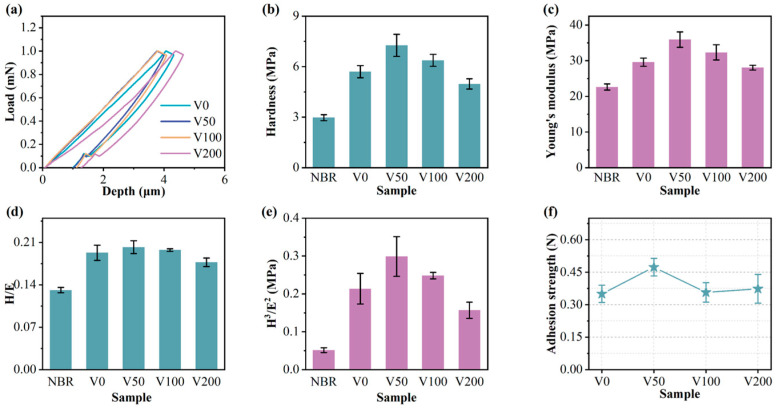
(**a**) Load–unloading curve of each sample; (**b**) hardness of each sample; (**c**) Young’s modulus of each sample; (**d**) H/E of each sample; (**e**) H^3^/E^2^ of each sample; (**f**) adhesion strength of each sample.

Figure 7a presents a distinct load-depth indentation profile for each sample. The indenter’s penetration into the specimen’s surface during the loading stage induces simultaneous elastic and plastic deformation. The subsequent unloading phase allows for elastic recovery, enabling the determination of both the hardness and elastic modulus. Figure 6b,c show the changes in hardness and Young’s modulus for NBR and N-DLC films on NBR substrates under varying bias voltages. Typically, hardness correlates with the area affected by the maximum indentation depth (h_max_), while Young’s modulus is linked to the observed changes in hardness [6]. After unloading, the indentation rebounds much deeper than the thickness of the DLC (about 180 nm). Thus, the hardness value represents the composite hardness of the film and substrate, calculated using the Oliver–Pharr method [27]. Initially, the film’s hardness and Young’s modulus increase and then decrease with increasing substrate bias. A peak hardness of 6.8 MPa was observed at a bias of −50 V. This may be attributed to the decline in the sp^3^ content of the film as the bias voltage increases, signifying more graphite bonds and fewer diamond-like bonds, thereby reducing the film’s hardness and elastic modulus. A higher substrate bias (>−150 V) also induces the formation of soft second phases in N-DLC, characterized by highly graphitized or nitrogen-rich carbon regions. These second phases diminish the mechanical properties and stability of the film. The values of H/E and H^3^/E^2^, depicted in Figure 7d,e, serve as indicators of the film’s resistance to crack formation and plastic deformation, respectively. These metrics are positively associated with the film’s wear resistance, suggesting that higher values denote enhanced durability [40,41]. With increasing bias voltage, the H/E and H^3^/E^2^ values initially increase before decreasing. This phenomenon is attributed to the suitable bias voltage facilitating a smooth transition between NBR and N-DLC, providing the V50 film with notable toughness and strong resistance to plastic deformation, thereby reducing the risk of brittle fracture.

### 3.5. Tribological Properties

**Figure 8:**
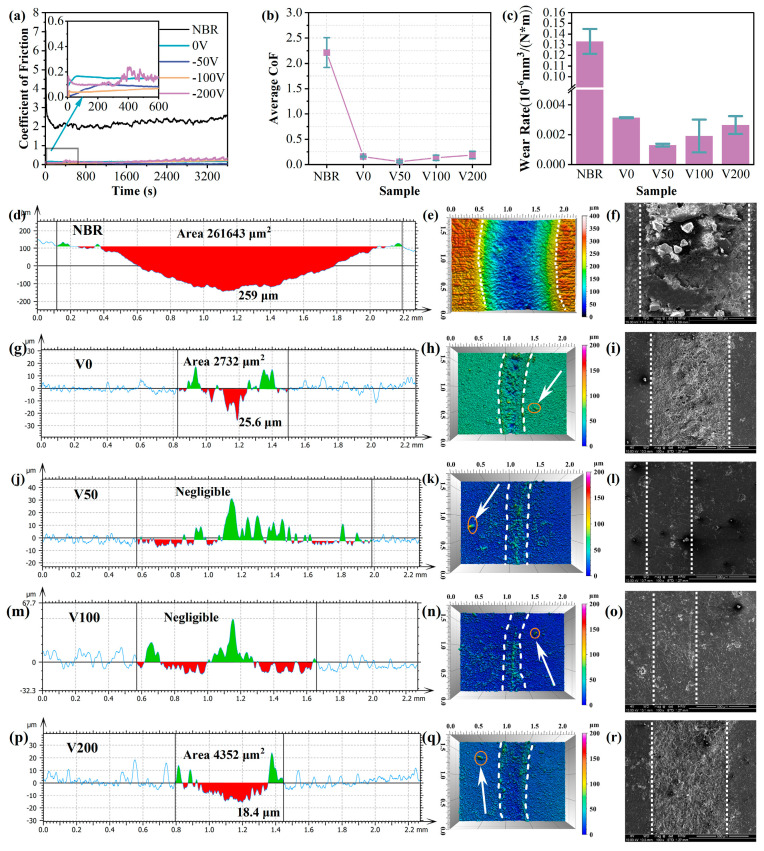
(**a**) Coefficient of friction of each sample; (**b**) average coefficient of friction of each sample; (**c**) wear rate of each sample. Wear depth cross-section and 3D wear trajectory morphology of N-DLC films on NBR with different bias voltages, NBR: (**d**–**f**); V0: (**g**–**i**); V50: (**j**–**l**); V100: (**m**–**o**); V200: (**p**–**r**).

To evaluate the tribological properties of N-DLC films, rotational friction tests were conducted on NBR-coated N-DLC samples across varying bias voltages, with the results depicted in Figure 8. In Figure 8a,b, the evolving trend of the CoF curve for each sample over time and its average CoF during the friction process are presented. It is apparent that despite displaying differing CoF values, all samples under varied bias voltages exhibit a significant reduction compared to the NBR matrix. The NBR rubber not only displays an average CoF as high as 2.2, but also demonstrates substantial fluctuations during the friction process, indicating poor wear resistance. Conversely, the modified N-DLC film markedly reduces the CoF, notably the V50 sample, boasting an average CoF as low as 0.05, highlighting its wear resistance. Moreover, as inferred from the wear rate (depicted in Figure 8c), compared to NBR, the wear rate was reduced by two orders of magnitude. Consequently, the V50 sample not only demonstrates good wear resistance, but also exhibits excellent durability.

The wear scar morphology of each sample, as illustrated in Figure 8d–r, reveals minimal wear for specimens V50 and V100. An adhesion strength analysis indicated that sample V50 exhibited the highest bonding strength, with a coefficient of friction (CoF) around 0.05, and the lowest wear rate, measured at 10^−9^. A three-dimensional wear trace analysis showed minor wear with visible debris for samples V0 and V200, as confirmed via SEM testing. In contrast, V50 and V100 displayed exceptional wear resistance, with no visible wear marks, aligning with the three-dimensional wear trace representation. The wear area on the V50’s surface was barely noticeable, featuring only occasional protrusions, likely surface impurities. In addition, a small number of bumps are vaguely visible beyond the wear marks in the three-dimensional wear trace diagram (pointed out by the white arrows in the diagram), which are attributed to impurity particles in the rubber substrate itself. Therefore, applying a moderate substrate bias can significantly improve the N-DLC film’s adhesion to the substrate, enhancing wear resistance under low-friction conditions and ensuring durability under continuous wear.

**Figure 9:**
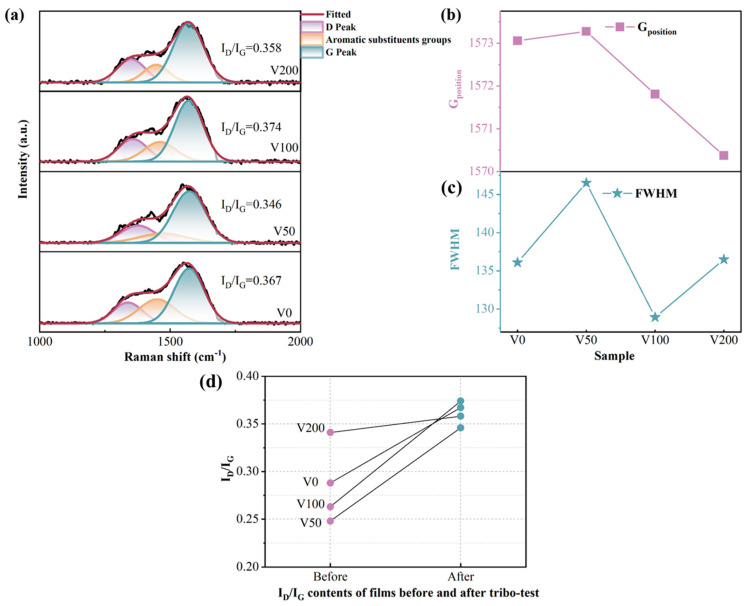
(**a**) Raman spectra of the wear marks of each sample; (**b**) G peak position of each sample; (**c**) FWHM of each sample; (**d**) I_D_/I_G_ ratio of each sample before and after the friction test.

Additionally, to examine the structural alterations at the wear scars, Raman spectroscopy was performed on these regions. Figure 9a depicts the fitting outcomes of the Raman spectra for each sample. Furthermore, an investigation into the peak position and FWHM of the Raman G peak at the wear scars was conducted. At a wear scar, the G peak position deviates to varying degrees from the standard G peak at 1580 cm^−1^, with a greater offset indicating higher internal stress [42]. The V50 sample still exhibited minimal internal stress, attributed to the effective combination of N-DLC prepared under a −50 V bias and NBR. Figure 9d illustrates the I_D_/I_G_ ratio for each sample before and after the friction test. A noticeable increase in the I_D_/I_G_ ratio is observed for all samples, suggesting that the samples experienced heating during the friction process, leading to distinct alterations [43]. With increasing graphitization, the graphite wear debris acts as a transfer layer and solid lubricant, resulting in a significant reduction in the CoF.

**Figure 10:**
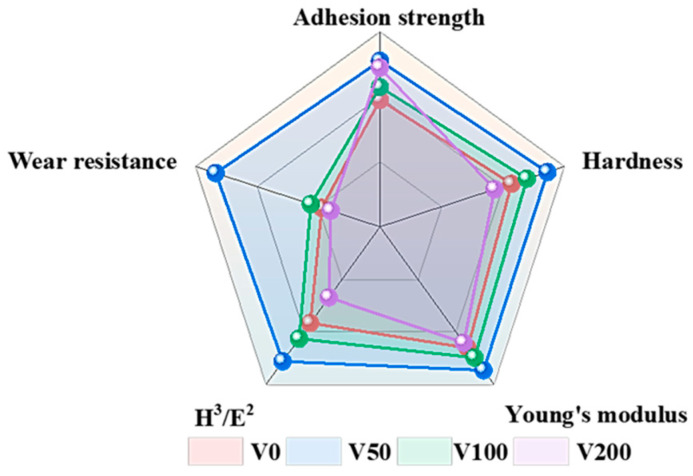
Comparison of mechanical properties between samples.

The mechanical properties of the film samples prepared under different bias pressures were compared, and comparison results were plotted in Figure 10. As illustrated in Figure 10, the mechanical properties of the V50 sample exceled in all aspects compared to those of other samples within the same group. It is noteworthy that the wear resistance index is derived by reciprocating the CoF.

## 4. Mechanism Discussion

**Figure 11:**
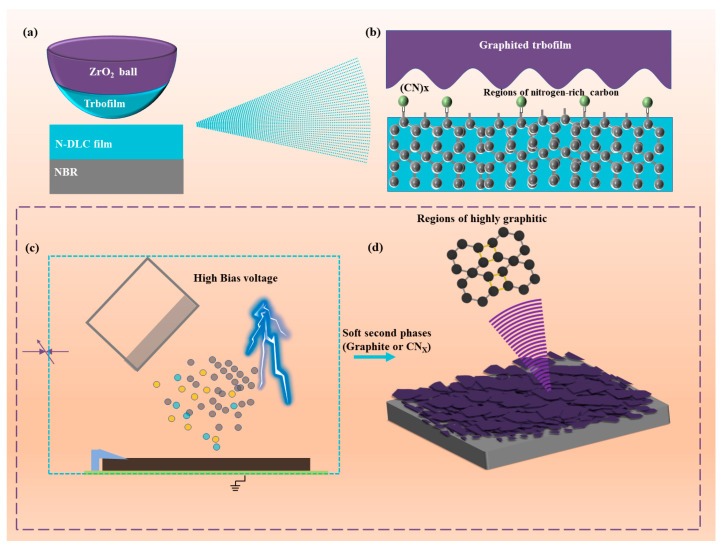
(**a**,**b**) Friction reduction mechanism of N-DLC film in air; (**c**,**d**) effect of high bias voltage on N-DLC film.

Figure 11 illustrates the N-DLC friction reduction mechanism and the impact of high bias voltage on N-DLC film performance, alongside experimental findings. Figure 11a,b depict the tribological mechanism of N-DLC in an air environment. Consistent with the Raman results at the wear marks, a consistent upward trend in the I_D_/I_G_ is observed after all samples undergo 1 h of rotational friction. This indicates the potential graphitization of N-DLC due to heat during friction, leading to the formation of a soft transfer film that improves rubber–film friction properties. Additionally, Figure 11c shows that a high bias voltage (>150 V) during magnetron sputtering readily induces the formation of a soft second phase on the film surface, represented as the (CN)x phase, in Figure 11b,d, and graphite phase [44]. This soft second phase contributes to N-DLC films under high bias voltages exhibiting a higher I_D_/I_G_ ratio and lower hardness, thereby reducing their tribological properties [45]. Consequently, in the preparation of N-DLC films, selecting an appropriate and moderate bias voltage is crucial for enhancing both mechanical and tribological properties.

## 5. Conclusions

In this investigation, the DC magnetron sputtering (DC-MS) method was utilized to coat NBR with N-DLC films, aiming to improve its tribological characteristics. The study assessed how varying bias voltages influenced the N-DLC films’ surface morphology, structural composition, wettability, and tribological performance. The following insights were derived from the research findings:

(1) The substrate bias voltage plays a pivotal role in determining the surface morphology, structural composition, and surface roughness of N-DLC films produced through DC-MS technology, thereby affecting their wettability, mechanical properties, and tribological performance. An increase in bias voltage results in the incorporation of softer second phases, such as (CN)x and graphite, into the N-DLC film. These softer constituents adversely impact the film’s mechanical and tribological properties.
(2) Moderate bias application was found to enhance the bonding strength between DLC and rubber, along with their tribological performances. At a bias of −50 V, N-DLC films exhibited commendable adhesion to the substrate and tribological characteristics. The coefficient of friction (CoF) remained consistently low at 0.052 throughout the friction testing, and the film showed no significant wear marks post experiment. However, the adhesion of NBR coated with N-DLC film was compromised under relatively high bias conditions during the deposition process.
(3) N-DLC films applied to NBR surfaces demonstrate enhanced tribological characteristics primarily due to increased hardness, improved adhesion between the film and substrate, reduced surface roughness, and diminished adhesion at the friction interface.

## Figures and Tables

**Table 1:** Process parameters for N-DLC film preparation under different bias voltages.

				Flow Rate (sccm)			
No.	Bias Voltage (V)	Press (Pa)	Power (W)	Ar	N_2_	Time (min)	Temperature (°C)
V0	0	1.4	100	30	3	60	25
V50	−50						
V100	−100						
V200	−200						

## Data Availability

The data presented in this study are available from the corresponding author upon request.
